# Weaknesses in the reporting of cross-sectional studies according to the STROBE statement: the case of metabolic syndrome in adults from Peru

**Published:** 2015-12-30

**Authors:** Jose Carlos Tapia, Eloy F Ruiz, Oscar J Ponce, German Malaga, Jaime Miranda

**Affiliations:** 1 Facultad de Medicina Alberto Hurtado, Universidad Peruana Cayetano Heredia Lima, Peru; 2 CRONICAS Centro de Excelencia en Enfermedades Crónicas, Universidad Peruana Cayetano Heredia. Lima, Peru; 3 CONEVID, Unidad de Conocimiento y Evidencia, Universidad Peruana Cayetano Heredia, Lima, Peru

**Keywords:** Metabolic syndrome, epidemiology, prevalence, adult, Peru

## Abstract

**Introduction:**

The inadequate reporting of cross-sectional studies, as in the case of the prevalence of metabolic syndrome, could cause problems in the synthesis of new evidence and lead to errors in the formulation of public policies.

**Objective:**

To evaluate the reporting quality of the articles regarding metabolic syndrome prevalence in Peruvian adults using the STROBE recommendations.

**Methods:**

We conducted a thorough literature search with the terms "Metabolic Syndrome", "Sindrome Metabolico" and "Peru" in MEDLINE/PubMed, LILACS, SciELO, LIPECS and BVS-Peru until December 2014. We selected those who were population-based observational studies with randomized sampling that reported prevalence of metabolic syndrome in adults aged 18 or more of both sexes. Information was analysed through the STROBE score per item and recommendation.

**Results:**

Seventeen articles were included in this study. All articles met the recommendations related to the report of the study's rationale, design, and provision of summary measures. The recommendations with the lowest scores were those related to the sensitivity analysis (8%, n= 1/17), participant flowchart (18%, n= 3/17), missing data analysis (24%, n= 4/17), and number of participants in each study phase (24%, n= 4/17).

**Conclusion:**

Cross-sectional studies regarding the prevalence of metabolic syndrome in peruvian adults have an inadequate reporting on the methods and results sections. We identified a clear need to improve the quality of such studies.

## Abstract (es)

**Introducción:**

El reporte inadecuado de estudios transversales, como en el caso de la prevalencia de síndrome metabólico, podría causar problemas en la síntesis de nueva evidencia y generar errores en la formulación de políticas públicas.

**Objetivo:**

Evaluar la calidad de reporte de estudios transversales sobre la prevalencia de síndrome metabólico en Perú utilizando las recomendaciones de STROBE.

**Métodos:**

Se realizó una búsqueda bibliográfica exhaustiva hasta Diciembre 2014 en MEDLINE/PubMed, LILACS, SciELO, LIPECS y BVS-Perú con los términos "Metabolic Syndrome", "Sindrome Metabolico" y "Peru". Se seleccionaron estudios observacionales con base poblacional, muestreo aleatorizado, que reportaran datos de prevalencia en adultos mayores de 18 años de ambos sexos. La información fue analizada a través de STROBE según puntuación por artículo y por recomendación.

**Resultados:**

Diecisiete artículos fueron incluidos en este estudio. Todos cumplieron con las recomendaciones relacionadas con el reporte de razones y fundamentos de la investigación, reporte del diseño de estudio y la proporción de medidas de resumen. Las recomendaciones con menor puntaje fueron las relacionadas a la descripción del análisis de sensibilidad (8%, n= 1/13), consideración del uso de diagrama de flujo para los participantes (18%, n= 3/17), explicación del análisis de datos ausentes (24%, n= 4/17) y del número de participantes en cada fase (24%, n= 4/17).

**Conclusión:**

Los estudios transversales sobre prevalencia de síndrome metabólico en adultos del Perú presentan un inadecuado reporte en las secciones de métodos y resultados. Se identifica una clara necesidad de mejorar la calidad de este tipo de estudios.

## Introduction 

The inadequate reporting of biomedical research is a longstanding and potentially serious global problem that is not entirely evident to many researchers 1. All scientific study must be fully and accurately reported, allowing a proper understanding of their methodology, findings and replication of the same if needed 2,3. However, most of the reports are far from those ideals 2. For this reason, many guidelines that seek to standardize and improve the reporting quality of different types of research were developed in the past few years 4. *Strengthening the Reporting of OBservational studies in Epidemiology* (STROBE) is a guideline whose recommendations have been established in order to adequately report observational studies (cohort, case-control and cross-sectional studies) 3,5. It should be noted that the STROBE recommendations assess the quality of reporting, but not the research or methodological quality *per se* 6.

Moreover, an inadequate reporting of cross-sectional studies could lead to problems in the synthesis and adoption of new evidence and generate errors in the justification and formulation of public policies 2, especially in regions with limited resources like Peru or other Latin American countries. For example, the prevalence of Metabolic Syndrome (MS) is relevant to public health issues because it had been associated with an increase of two to three times the risk of presenting a heart attack or stroke 7 and five times higher risk of developing type 2 diabetes mellitus 8,9. Nonetheless, the diversity of criteria for defining MS 10-16 associated with the poor reporting in cross-sectional studies, generate confusion when interpreting the real extent of the problem. For that reason, this study aims to evaluate the reporting quality of cross-sectional studies regarding the prevalence of MS in Peruvian adults, using the STROBE recommendations as an objective tool.

## Materials and Methods

This descriptive study was carried out in two stages. First, a systematic literature search was performed to identify the articles to be included in the study. Then, the quality of the reporting was assessed using STROBE. This study followed the recommendations of the PRISMA (Preferred Reporting Items for Systematic Reviews and Meta-Analyses) statement for its reporting 17.

### Search strategy

We conducted the search in MEDLINE/PubMed (1997 - 4/12/2014), LILACS (1982 - 4/12/2014), SciELO (1999 - 4/12/2014), LIPECS (1987 - 4/12/2014) and BVS-Perú (INS, MINSA and OPS; 1997 - 4/12/2014) after reaching consensus regarding each database search strategy. The terms "Metabolic Syndrome" and "Sindrome Metabolico" were used in combination with the term "Peru" depending upon the database. The terms were used in english for MEDLINE via PubMed; in Spanish for LIPECS, BVS-Perú and OPS; or in both languages for LILACS and SciELO. The last search was completed on December 4, 2014. This was done simultaneously and independently by three researchers (JCT, EFR, OJP) and a list of found items was made. Then, search results were compared and no differences in the outcome were found among the three authors. 

### Study selection

The selection was made with the purpose of obtaining studies whose external validity allow us to extrapolate their results to different populations of Peru. The following eligibility criteria were defined: 1) population-based observational studies; 2) studies involving random sampling instead of volunteer recruitment; 3) studies that reported prevalence data of MS according to a selected criteria and 4) studies involving adults 18 years or older of both sexes. There were no language restrictions. Full-text articles were evaluated by three researchers (JCT, EFR, OJP) and those who met the inclusion criteria were selected. Additionally, a secondary search through the bibliographical references of the selected articles was made and duplicates were removed. We excluded studies involving pediatric or inpatient population, workers from any institution or patients recruited through health campaigns. Reviews, editorials or short communications were also excluded. A fourth investigator (GM) was consulted in the event of discrepancies and reached a consensus.

### Instrument

We used the STROBE recommendations as an objective tool to evaluate the quality of the reports. STROBE presents 32 recommendations for the appropriate reporting of observational studies. These recommendations describe the proper way of reporting the title, abstract, introduction, methods, results, discussion and financing 5. According to the language of each publication, we used the cross-sectional studies suggested-version, available in English 3 or Spanish 18.

For this study, we used 30 of the 32 recommendations from cross-sectional studies. We considered as not-applicable the items 16b (the limits of the ranges for continuous variables of MS -blood pressure, glucose, HDL, triglycerides, or abdominal circumference are already defined for each criterion) and 16c (the objectives of the studies were not to evaluate the report of relative or absolute risk). Additionally, two recommendations were considered as not-applicable for the following cases: 12d if the study had a simple random sampling (single-stage) and 12e if the article fulfilled 12a, 12b and 12c recommendations.

### Data extraction

Two formats for data extraction were developed. The first contained information about the general characteristics of each article: first author, publication year, name of the study from which the data came from, publication language, study period, city, type of population, sampling type, age range, sample size and criteria used to define MS. The second format is a list with 30 of the 32 STROBE recommendations.

Three researchers (JCT, EFR, OJP) reviewed the full-text of the articles with their respective protocols, in case the latter were cited, and the data was extracted. Each investigator assessed whether the reports identified met or not the STROBE recommendations. We did not hide the names of the authors nor the title of the articles.

Finally, the corresponding author of each included article was contacted by email. In each email, we presented the purpose of the study and the recommendations fulfilled by the article, based on our analysis according to STROBE. This step was done in order to clarify potential disagrements with our assessment. The inputs of each author were analyzed according to the methodology described above and modifications to our analysis were performed as appropriate. In case of no response, a reminder was sent 7 days after the first email. We waited in total for 14 days for the author to reply, otherwise our analysis was considered as the final result. 

### Analysis

Two types of scores were reported as follows: score per article and per recommendation. The score per article was defined as the number of the STROBE recommendations adequately reported, divided by the total of recommendations applicable per article and expressed as a percentage. The score per recommendation was defined as the number of articles that met each STROBE recommendation, divided by the total of articles for which the recommendation were applicable and expressed as a percentage.

## Results

###  Results of the literature search

**Figure 1.**
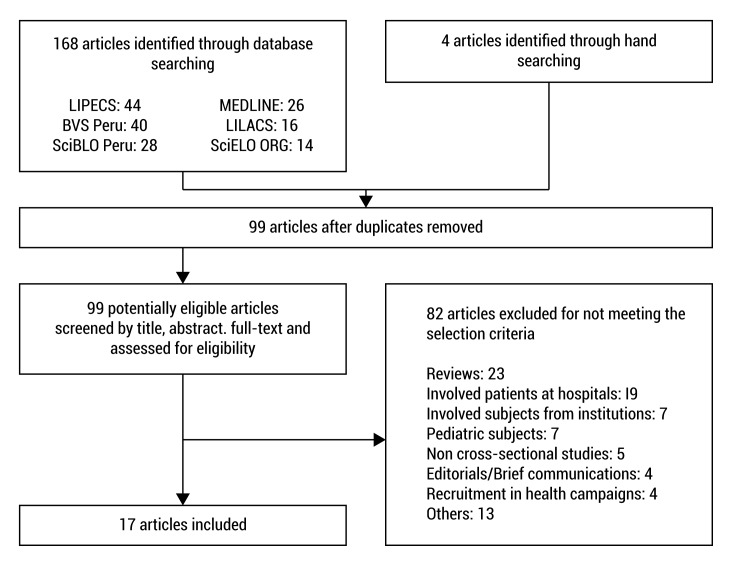
Flowchart of the selection of articles

We found 168 articles through the database search and 4 through the secondary search within the references of the included articles. From the 172 articles, 73 were excluded for being duplicates and the remaining 99 were examined in full-text. Of these, 82 were discarded because they did not meet the selection criteria, resulting in a total of 17 articles for data extraction 19-35 (Fig. 1).

### General features of the reports

Table 1 summarizes the main features of the 17 included articles. Four articles 19,20,23,24 were published in 3 Peruvians journals and 13 21,22,24-33,35 in 11 foreign journals. Of the total, 3 reported belonging to the ENINBSC study 36, 3 to the PERU MIGRANT study 37, 2 to the CARMELA study 33, 2 to the PREVENCION study 38, 2 to the PIRS study and 1 to the FRENT study. These population-based studies were conducted between 1999 and 2008 and involved 12,789 participants from different Peruvian cities, including urban and rural population, and rural-urban migrants. According to the sampling strategy, 4 had simple random sampling (single-stage), while the 13 remaining had multistage randomized sampling. In addition, 12 reports were published in English and 5 in Spanish. Finally, regarding the criteria used to determine prevalence of MS in adults from Peru, 7 articles (41%) used the NCEP-ATP III criteria 12, 2 the JIS criteria 16, 1 the AHA/NHLBI criteria 13, 1 the IDF criteria 14, and the others used more than one criterion.

**Table 1.** Main features of the articles regarding the prevalence of Metabolic Syndrome of Adults in Peru.

Reference	Article	Publication Year	Protocol	Publication language	Study Period	City	Population	Sampling Type	Age	Sample Size	MS Criteria
Soto *et al* ^19^	A-1	2005	NS	Spanish	2004	Lambayeque	Urban, rural	Randomized, multistage, stratified and by clusters	30 - 70	1,000	NCEP-ATP III^12^, ILIBLA
Guarnizo *et al* ^20^	A-2	2006	NS	Spanish	2004 - 2005	Lambayeque	Urban, rural	Randomized, multistage, stratified and by clusters	30 - 70	621	NCEP-ATP III^12^, IDF^14^, ILIBLA
Lorenzo *et al* ^21^	A-3	2006	PIRS	English	1999 - 2001	Lima	Urban	Randomized, multistage, stratified and by clusters	35 - 64	346	rNCEP-ATP III^15^, IDF^14 ^
Seclén *et al* ^22^	A-4	2006	PIRS	English	1999 - 2001	Lima	Urban	Randomized, multistage, stratified and by clusters	≥30	612	NCEP-ATP III^12 ^
Pajuelo *et al* ^23^	A-5	2007	ENINBSC	Spanish	2004 - 2005	National	Urban, rural	Randomized, multistage, stratified and by clusters	≥20	4,091	NCEP-ATP III^12 ^
Medina-Lezama *et al* ^24^	A-6	2007	PREVENCION	English	2004 - 2006	Arequipa	Urban	Randomized, multistage, stratified and by clusters	20 - 80	,1878	NCEP-ATP III^12^, AHA/NHLBI^13 ^
Baracco *et al* ^25^	A-7	2007	NS	English	2002 - 2003	Lima,	Urban, rural	Simple randomization	≥30	271	NCEP-ATP III^12 ^
Schargrodsky *et al* ^26^	A-8	2008	CARMELA	English	2003 - 2005	Lima	Urban	Randomized, multistage and stratified	25 - 64	1,652	NCEP-ATP III^12 ^
Cárdenas *et al* ^27^	A-9	2009	ENINBSC	Spanish	2004 - 2005	National	Urban, rural	Randomized, multistage, stratified and by clusters	≥20	4,053	IDF^14^
Escobedo *et al* ^28^	A-10	2009	CARMELA	English	2003 - 2005	Lima	Urban	Randomized, multistage and stratified	25 - 64	1,645	NCEP-ATP III^12 ^
Gelaye et al^29^	A-11	2009	FRENT Study	English	2006	Lima,	Urban	Randomized, multistage and stratified	≥18	1,675	NCEP-ATP III^12 ^
Masterson* et al* ^30^	A-12	2010	PERU MIGRANT	English	2007 - 2008	Lima,	Rural, urban, Urban, rural, rural-urban migrants	Simple randomization	≥30	985	AHA/NHLBI^13 ^
Medina-Lezama *et al* ^31^	A-13	2010	PREVENCION	English	2004 - 2006	Arequipa	Urban	Randomized, multistage, stratified and by clusters	20 - 80	1448	AHA/NHLBI^13^, JIS^16^
Miranda *et al* ^32^	A-14	2011	PERU MIGRANT	English	2007 - 2008	Lima,	Rural, urban Urban, rural, rural-urban migrants	Simple randomization	≥30	989	JIS^16^
Boissonet *et al* ^33^	A-15	2011	CARMELA	English	2003 - 2005	Lima	Urban	Randomized, multistage and stratified	25 - 64	1652	NCEP-ATP III^12 ^
Pajuelo *et al* ^34^	A-16	2012	ENINBSC	Spanish	2004 - 2005	<1000,	Urban, rural	Randomized, multistage, stratified and by clusters	≥20	3384	NCEP-ATP III^12 ^
Bernabe-Ortiz *et al* ^35^	A-17	2012	PERU MIGRANT	English	2007 - 2008	Lima	Rural-urban Migrants	Simple randomization	≥30	589	JIS^16^

MS: Metabolic Syndrome NS: Not stated, NCEP-ATP III: National Cholesterol Education Program-Adult Treatment Panel III; ILIBLA: International Lipid Information Bureau-Latin America; IDF: International Diabetes Federation; rNCEP-ATP III: revised NCEP-ATP III; AHA/NHLBI: American Heart Association/National Heart, Lung and Blood Institute; JIS: Joint Interim Statement

### Reporting Quality according to the STROBE recommendations

Thirteen (76%) out of the 17 corresponding authors answered the emails, commenting and supporting if they agreed (5/13) or disagreed (8/13) with our analysis. Most of the disagreements were found in the recommendations related to the statistical analysis (sample size modification according to the sampling strategy, and the analysis of sensitivity, subgroups and missing data) and the description of the number of participants for each study phase. Based on these disagreements, each recommendation was reviewed again and answers were sent via email with the respective modifications.

Table 2 shows the number of articles that met each STROBE recommendation. The recommendations that were fully met were those related to the reporting of the reasons and rationale of the investigation (recommendation 2), to the reporting of the study design (recommendation 4) and to provide summary measures (recommendation 15). In addition, 16 out of the 17 articles adequately defined their variables and their respective measurement methods (recommendation 7 and 8). They also reported how they grouped and analyzed the quantitative variables (recommendation 11) and provided confidence intervals for their estimates (recommendation 16a). On the other hand, the recommendations with the lowest scores were those related to the description of the sensitivity analysis (recommendation 12e; 1/13 (8%)), to consider the use of a flowchart for the participants (recommendation 3; 3/17 (18%)), to explain the analysis of the missing data (recommendation 12c; 4/17 (24%)), to specify the number of participants in each study phase (recommendation 13a; 4/17 (24%)), to describe the reasons for the loss of the participants (recommendation 13b; 5/17 (29%)) and to specify the steps taken to identify possible sources of bias (recommendation 9; 7/17 [41%]). In supplementary material is shown the score assigned for each STROBE recommendation applicable per article (supplementary material). Only 3 out of the 17 articles met all the STROBE recommendations.

**Table 2.** Number of articles that fulfill each recommendation of the STROBE Statement.

Section	Subsection	Code	Recommendation	Articles that fulfill each STROBE recommendation
				n(%)
Title and abstract	Title and abstract	1a	Indicate the study's design with a commonly used term in the title or the abstract	13 (76)
		1b	Provide in the abstract an informative and balanced summary of what was found	15 (88)
Introduction	Background/rationale	2	Explain the scientific background and rationale for the investigation being reported.	17 (100)
	Objectives	3	State specific objectives, including any prespecified hypotheses.	13 (76)
Methods	Study design	4	Present key elements of study design early in the paper.	17 (100)
	Setting	5	Describe the setting, locations, and relevant dates, including periods of recruitment, exposure, follow-up, and data collection.	10 (59)
	Participants	6	Cross-sectional study: give the eligibility criteria, and the sources and methods of selection of participants.	17 (100)
	Variables	7	Clearly define all outcomes, exposures, predictors, potential confounders, and effect modifiers. Give diagnostic criteria, if applicable.	16 (94)
	Data sources/measurement	8	For each variable of interest, give sources of data and details of methods of assessment (measurement). Describe comparability of assessment methods if there is more than one group.	16 (94)
	Bias	9	Describe any efforts to address potential sources of bias.	7 (41)
	Study size	10	Explain how the study size was arrived at.	9 (53)
	Quantitative variables	11	Explain how quantitative variables were handled in the analyses. If applicable, describe which groupings were chosen and why.	16 (94)
	Statistical methods	12a	Describe all statistical methods, including those used to control for confounding.	15 (88)
		12b	Describe any methods used to examine subgroups and interactions.	15 (88)
		12c	Explain how missing data was addressed.	4 (24)
		12d	Cross-sectional study: If applicable, describe analytical methods taking account of sampling strategy.	7 (41)
		12e	Describe any sensitivity analyses.	1 (6)
Results	Participants	13a	Report numbers of individuals at each stage of study-eg numbers potentially eligible, examined for eligibility, confirmed eligible, included in the study, completing follow-up, and analysed	4 (24)
		13b	Give reasons for non-participation at each stage.	5 (299
		13c	Consider use of a flow diagram.	3 (18)
	Descriptive data	14a	Give characteristics of study participants (eg. Demographic, clinical, social) and information on exposures and potential confounders.	13 (76)
		14b	Indicate number of participants with missing data for each variable of interest.	3 (18)
	Outcome data	15	Cross-sectional study: report numbers of outcome events or summary measures.	17 (100)
	Main results	16a	Give unadjusted estimates and , if applicable, confounder-adjusted estimates and their precision (eg. 95% confidence interval). Make clear which confounders were adjusted for and why they were included.	16 (94)
		16b	Report category boundaries when continuous variables were categorized.	NA
		16c	If relevant, consider translating estimates of relative risk into absolute risk for a meaningful time period.	NA
	Other analyses	17	Report other analyses done - eg. analyses of subgroups and interactions, and sensitivity analyses.	15 (85)
Discussion	Key results	18	Summarize key results with reference to study objectives.	16 (94)
	Limitations	19	Discuss limitations of the study, taking into account sources of potential bias or imprecision. Discuss both direction and magnitude of any potential bias.	9 (53)
	Interpretation	20	Give a cautious overall interpretation of results considering objectives, limitations, multiplicity of analyses, results from similar studies, and other relevant evidence.	14 (82)
	Generalisability	21	Discuss the generalizability (external validity) of the study results.	13 (76)
Other information	Funding	22	Give the sources of funding and the role of the funders for the present study and, if applicable, for the original study on which present article is based.	13 (76)

## Discussion

The results of the analysis show that the fulfillment of the STROBE recommendations for the reporting of cross-sectional studies on the prevalence of MS was mixed, being the ones related to the methodology and results the lowest. These deficiencies are particularly critical for methodologically well-conducted studies and properly analyzed. For that reason, every scientific report, to be reliable, must provide a clear, complete and transparent presentation of what was planned, made and found, in order to facilitate the adequate interpretation and diffusion of their findings 3. 

Of the 17 articles involving 12,789 people, 14 (11,804 people) showed limitations when reporting the methodology related to the statistical analysis, including sensitivity analysis, missing data and sources of bias. Also, the report of the results was not clear regarding the description of the participants flow and the reasons of loss in each study phase. These weaknesses are not unique to this scenario as further investigations, in fields other than MS, have used the recommendations of STROBE to analyze the report of other observational studies and had also described limitations in the same areas 39-45. On the other hand, 7 (41%) out of the 17 articles were published before the creation of STROBE and this could explain their weaknesses in its reporting in contrast to those published after (average score pre-STROBE 60%; average score post-STROBE: 77%). However, it has been shown that the quality of reporting of observational studies still remains suboptimal several years after the creation of STROBE 46 and that, despite some authors refer to follow the recommendations of STROBE, more than half do it inappropriately 6. Still, 3 articles (985 people) 30,32,35 mentioned using STROBE to write its report and when analyzed, it was certified that they met all recommendations.

The elaboration of an optimal research report is responsibility of the main authors, but several mechanisms (editorial policies editorial board, external reviewers) play a role in the process of publishing and also aim to an appropriate report 47. In order to achieve this objective, biomedical journals should adhere to reporting guidelines such as STROBE; also, editors and reviewers should be trained to demand its proper use 2. In the case of biomedical journals in Latin America and the Caribbean, only 28% recommends on its website any specific guideline to improve the quality of reporting 47. In this instance, although we have no data regarding the editorial policies of the journals at the time of publication of the selected articles, currently 43% of the 14 journals (2 of 3 peruvian journals and 4 of 11 foreign journals) suggest the authors to follow the STROBE recommendations as an editorial policy. The quality of reporting of observational and cross-sectional studies could be improved if journals introduce an active policy of adherence to reporting guidelines such as STROBE 46,48. Furthermore, an open access initiative that seeks to spread this kind of reporting guidelines is the EQUATOR Network 49 (http://www.equator-network.org/). 

Even though there are barriers to provide more details of what has been done and found, such as the article's length allowed in biomedical journals or the cost of its publication, the prior publication of the study protocol or the publishing of supplementary data online (eg. https://figshare.com/) are some alternatives to overcome these limitations.

Considering the above, it should be noted that the scientific reports play a greater purpose in addition to the generation of new knowledge. In particular, epidemiological studies have different audiences, uses and implications. For a more technical audience, studies should report detailed estimates of the burden of the diseases that allow prioritization of public policies. On the other hand, in case of a more general audience, they should provide a consistent message about a particular situation. On both platforms, technical and general audiences, we found that the articles analyzed regarding MS in Peru have significant restrictions in its report that limit the appropriate use of their findings.

The limitations of our study are that some of our results might have been different if they were assesed by other researchers, however, to avoid subjective decisions, each corresponding author was contacted to verify our analysis, obtaining a high rate response (76%). Furthermore, we can not assume that every STROBE recommendation has the same impact on the quality of the report, thus assigning an equitable score to each recommendation could be considered as arbitrary. However, we decided to use this strategy to help readers have a global view of the quality of the reports.

## Conclusion

Cross-sectional studies on the prevalence of MS in peruvian adults show an inadequate reporting of important areas such as methods and results. This finding identifies a clear need to improve the reporting quality of such studies in order to fulfill its role to adequately inform relevant subjects for the implementation of public health policies. 

## Electronic supplementary material

Table s1. Recommendations from the STROBE Statement fulfilled by each article.
