# Supplementary material for: Weaknesses in the reporting of cross-sectional studies according to the STROBE statement: the case of metabolic syndrome in adults from Peru
Source: Colomb Med (Cali). 2015 Dec 30;46(4):168–75. (PMC4732506)
Supplement: Supplementary file 1 [file 1657-9534-cm-46-04-00168-s1.pdf]

Table s1: Recommendations from the STROBE Statement fulfilled by each article.

Recommendation			Article*																
			A-1[19]	A-2[20]	A-3[21]	A-4[22]	A-5[23]	A-6[24]	A-7[25]	A-8[26]	A-9[27]	A-10[28]	A-11[29]	A-12[30]	A-13[31]	A-14[32]	A-15[33]	A-16[34]	A-17[35]
Title and abstract																			
Title and abstract	1	a	1	1	0	1	0	1	1	1	1	1	1	1	0	1	1	0	1
		b	1	0	1	1	0	1	1	1	1	1	1	1	1	1	1	1	1
	Subtotal		2-Feb	1-Feb	1-Feb	2-Feb	0	2-Feb	2-Feb	2-Feb	2-Feb	2-Feb	2-Feb	2-Feb	1-Feb	2-Feb	2-Feb	1-Feb	2-Feb
			-100%	-50%	-50%	-100%	0%	-100%	-100%	-100%	-100%	-100%	-100%	-100%	-50%	-100%	-100%	-50%	-100%
Introduction																			
Background/rationale	2		1	1	1	1	1	1	1	1	1	1	1	1	1	1	1	1	1
Objetives	3		1	0	0	1	0	0	1	1	1	1	1	1	1	1	1	1	1
	Subtotal		2-Feb	1-Feb	1-Feb	2-Feb	1-Feb	1-Feb	2-Feb	2-Feb	2-Feb	2-Feb	2-Feb	2-Feb	2-Feb	2-Feb	2-Feb	2-Feb	2-Feb
			-100%	-50%	-50%	-100%	-50%	-50%	-100%	-100%	-100%	-100%	-100%	-100%	-100%	-100%	-100%	-100%	-100%
Métods																			
Study design	4		1	1	1	1	1	1	1	1	1	1	1	1	1	1	1	1	1
Setting	5		1	1	1	0	0	1	1	0	0	0	1	1	1	1	0	0	1
Participants	6		1	1	1	1	1	1	1	1	1	1	1	1	1	1	1	1	1
Variables	7		1	0	1	1	1	1	1	1	1	1	1	1	1	1	1	1	1
Data sources/measurement	8		1	0	1	1	1	1	1	1	1	1	1	1	1	1	1	1	1
Bias	9		0	0	0	0	1	0	0	0	1	0	1	1	0	1	0	1	1
Study size	10		1	1	0	1	1	0	0	0	1	0	0	1	0	1	0	1	1
Quantitative variables	11		1	0	1	1	1	1	1	1	1	1	1	1	1	1	1	1	1
		a	0	0	1	1	1	1	1	1	1	1	1	1	1	1	1	1	1
		b	0	0	1	1	1	1	1	1	1	1	1	1	1	1	1	1	1
Statistical methods	12	c	0	0	0	0	0	0	0	0	0	0	1	1	0	1	0	0	1
		d	0	0	0	0	1	1	NA	1	1	1	0	NA	0	NA	1	1	NA
		e	0	0	1	0	0	0	0	0	0	0	NA	NA	0	NA	0	0	NA
Subtotal			Jul-13	Apr-13	Sep-13	Aug-13	Oct-13	Sep-13	8-Dec	Aug-13	Oct-13	Aug-13	10-Dec	11-Nov	Aug-13	11-Nov	Aug-13	Oct-13	11-Nov
			-54%	-31%	-69%	-62%	-77%	-69%	-66%	-62%	-77%	-62%	-83%	-100%	-62%	-100%	-62%	-77%	-100%
Results																			
		a	0	1	0	0	0	0	0	0	0	0	0	1	0	1	0	0	1
Participants	13	b	0	1	0	0	0	0	1	0	0	0	0	1	0	1	0	0	1
		c	0	0	0	0	0	0	0	0	0	0	0	1	0	1	0	0	1
Descriptive data	14	a	1	1	1	1	0	1	1	1	0	0	1	1	1	1	1	0	1
		b	0	0	0	0	0	0	0	0	0	0	0	1	0	1	0	0	1
Outcome data	15		1	1	1	1	1	1	1	1	1	1	1	1	1	1	1	1	1
		a	1	1	1	1	0	1	1	1	1	1	1	1	1	1	1	1	1
Main results	16	b	NA	NA	NA	NA	NA	NA	NA	NA	NA	NA	NA	NA	NA	NA	NA	NA	NA
		c	NA	NA	NA	NA	NA	NA	NA	NA	NA	NA	NA	NA	NA	NA	NA	NA	NA
Other analyses	17		0	1	1	1	0	1	1	1	1	1	1	1	1	1	1	1	1
	Subtotal		3-Aug	6-Aug	4-Aug	4-Aug	1-Aug	4-Aug	5-Aug	4-Aug	3-Aug	3-Aug	4-Aug	8-Aug	4-Aug	8-Aug	4-Aug	3-Aug	8-Aug
			-38%	-75%	-50%	-50%	-13%	-50%	-63%	-50%	-38%	-38%	-50%	-100%	-50%	-100%	-50%	-38%	-100%
Discussion																			
Key results	18		1	1	1	1	0	1	1	1	1	1	1	1	1	1	1	1	1
Limitations	19		0	0	1	0	0	1	0	1	0	0	1	1	1	1	1	0	1
Interpretation	20		0	0	1	1	0	1	1	1	1	1	1	1	1	1	1	1	1
Generalizability	21		1	0	1	1	0	1	1	1	0	1	1	1	1	1	1	0	1
	Subtotal		2-Apr	1-Apr	4-Apr	3-Apr	0/0	4-Apr	3-Apr	4-Apr	2-Apr	3-Apr	4-Apr	4-Apr	4-Apr	4-Apr	4-Apr	2-Apr	4-Apr
			-50%	-25%	-100%	-75%	0%	-100%	-75%	-100%	-50%	-75%	-100%	-100%	-100%	-100%	-100%	-50%	-100%
Other information																			
Funding	22		1	1	1	1	0	1	0	1	1	0	1	1	1	1	1	0	1
	Subtotal		1-Jan	1-Jan	1-Jan	1-Jan	0/0	1-Jan	0/0	1-Jan	1-Jan	0/0	1-Jan	1-Jan	1-Jan	1-Jan	1-Jan	0/0	1-Jan
			-100%	-100%	-100%	-100%	0%	-100%	0%	-100%	-100%	0%	-100%	-100%	-100%	-100%	-100%	0%	-100%
	Total		17/30	14/30	20/30	20/30	Dec-30	21/30	20/29	21/30	20/30	18/30	23/29	28/28	20/30	28/28	21/30	18/30	28/28
			-57%	-47%	-67%	-67%	-40%	-70%	-69%	-70%	-67%	-60%	-79%	-100%	-67%	-100%	-70%	-60%	-100%

*References from each article are describe in the manuscript.
